# Apigenin Protects Endothelial Cells from Lipopolysaccharide (LPS)-Induced Inflammation by Decreasing Caspase-3 Activation and Modulating Mitochondrial Function

**DOI:** 10.3390/ijms140917664

**Published:** 2013-08-28

**Authors:** Silvia Duarte, Daniel Arango, Arti Parihar, Patrice Hamel, Rumana Yasmeen, Andrea I. Doseff

**Affiliations:** 1 Department of Molecular Genetics, the Ohio State University, 484 West 12th Avenue, Columbus, OH 43210, USA; E-Mails: duartesanmiguel.1@osu.edu (S.D.); Arango-tamayo.1@osu.edu (D.A.); asp1959@gmail.com (A.P.); hamel.16@osu.edu (P.H.); yasmeen.2@osu.edu (R.Y.); 2 Department of Internal Medicine, Division of Pulmonary, Allergy, Critical Care and Sleep, the Ohio State University, 473 West 12th Avenue, Columbus, OH 43210, USA; 3 The Heart and Lung Research Institute, the Ohio State University, 473 West 12th Avenue, Columbus, OH 43210, USA; 4 Molecular, Cellular and Development Biology Graduate Program, the Ohio State University, 333 West 10th Avenue, Columbus, OH 43210, USA; 5 Department of Biological Sciences, Government Postgraduate College of Excellence, Vikram University, Dashehra Maidan, Ujjain 456010, MP, India; 6 Department of Molecular and Cellular Biochemistry, the Ohio State University, 1645 Neil Avenue, Columbus, OH 43210, USA

**Keywords:** inflammation, flavonoids, metabolism, mitochondrial dysfunction

## Abstract

Acute and chronic inflammation is characterized by increased reactive oxygen species (ROS) production, dysregulation of mitochondrial metabolism and abnormal immune function contributing to cardiovascular diseases and sepsis. Clinical and epidemiological studies suggest potential beneficial effects of dietary interventions in inflammatory diseases but understanding of how nutrients work remains insufficient. In the present study, we evaluated the effects of apigenin, an anti-inflammatory flavonoid abundantly found in our diet, in endothelial cells during inflammation. Here, we show that apigenin reduced lipopolysaccharide (LPS)-induced apoptosis by decreasing ROS production and the activity of caspase-3 in endothelial cells. Apigenin conferred protection against LPS-induced mitochondrial dysfunction and reestablished normal mitochondrial complex I activity, a major site of electron leakage and superoxide production, suggesting its ability to modulate endothelial cell metabolic function during inflammation. Collectively, these findings indicate that the dietary compound apigenin stabilizes mitochondrial function during inflammation preventing endothelial cell damage and thus provide new translational opportunities for the use of dietary components in the prevention and treatment of inflammatory diseases.

## 1. Introduction

Sepsis is characterized by exacerbated inflammation and deregulated immune function leading to cardiac dysfunction and organ failure [1,2]. Sepsis affects 500,000 people in USA alone and has high mortality rates of up to 60%. Lack of current therapies is attributed to the failure of known interventions and limited understanding of the disease. Thus, the identification of new therapeutic and preventive approaches is crucial [3].

Increased inflammation induced by lipopolysaccharide (LPS) leads to inflammatory cytokine expression, inducing monocyte survival and neutrophil migration to sites of inflammation [4,5]. In addition, LPS promotes endothelial cell apoptosis [6], increases reactive oxygen species (ROS) production and induces mitochondrial dysfunction [7,8], contributing to the morbidity and mortality associated with sepsis [9]. Alterations of ROS and mitochondrial respiration affect metabolic function and have been involved in the pathogenesis of sepsis [8,10]. A causative link has been reported between sepsis-induced mortality and extensive mitochondrial damage, using muscle biopsies of critically ill septic patients [11]. In addition, animal studies showed impairment of cardiac mitochondria in sepsis [12]. It has been suggested that elevated ROS levels contributes to mitochondrial oxidative stress [13,14], and studies using mitochondria-targeted anti-oxidants showed improved outcomes in animal models [15,16]. Thus, reestablishment of metabolic balance may contribute to positive outcomes in sepsis.

Flavonoids are naturally occurring polyphenolic dietary compounds broadly found in fruits and vegetables, and constitute the largest class of nutraceuticals in our diet [17]. Apigenin is an anti-inflammatory dietary flavonoid, associated with lower prevalence of cardiovascular diseases [18]. Previously we reported that apigenin exerts anti-inflammatory activity *in vitro* and *in vivo* by modulating NF-κB activity, reducing inflammatory cytokine production in LPS-treated mice [19]. In addition, apigenin reduced neutrophil migration towards inflammatory microenvironments [5]. In general, flavonoids exist in plants linked to sugars and referred as glucosides [20,21]. For many glucoside flavonoids, brush border β-glucosidase activity in the small intestine is sufficient to hydrolyze the sugars, enabling absorption of the aglycones (flavonoid without the sugar, [22]). However, intestinal β-glucosidase is unable to cleave oligosaccharide moieties, and despite colonic bacteria cleaving these bonds, absorption is markedly decreased [23,24]. To overcome the poor absorption of flavonoids, we recently reported the development of apigenin-rich food formulations that increased absorption *in vivo*, reaching concentrations in serum similar to apigenin levels used in cellular studies [20]. Importantly, these aglycone apigenin-rich foods localized to mitochondria and reduced LPS-induced NF-κB activity and TNFα production in mouse macrophages [20,25]. Formulation of these nutraceutical diets with improved bioavailability might offer new opportunities for clinical interventions with flavonoids. Recently, we identified the direct targets of apigenin, that include among others, isocitrate dehydrogenase 3 (IDH3), a mitochondrial enzyme involved in the TCA cycle, and other metabolic enzymes [26]. However, despite these advances, the mechanisms underlying the anti-inflammatory activity of apigenin are yet to be dissected.

Here, we evaluated the effects of apigenin in endothelial cells during LPS-induced inflammation. We found that apigenin reduces LPS-induced endothelial cell apoptosis by decreasing the activity of caspase-3, a central modulator of apoptosis. Moreover, we found that apigenin reduced LPS-induced ROS production and restored mitochondrial function by modulating respiratory chain Complex I activity. Collectively these findings suggest that impaired metabolic functions characteristic of sepsis and other inflammatory diseases can be modulated by apigenin, suggesting a potential preventive and therapeutic value of phytochemicals in inflammatory diseases.

## 2. Results and Discussion

### 2.1. Protective Role of Apigenin against LPS-Induced Cell Death

**Figure 1:**
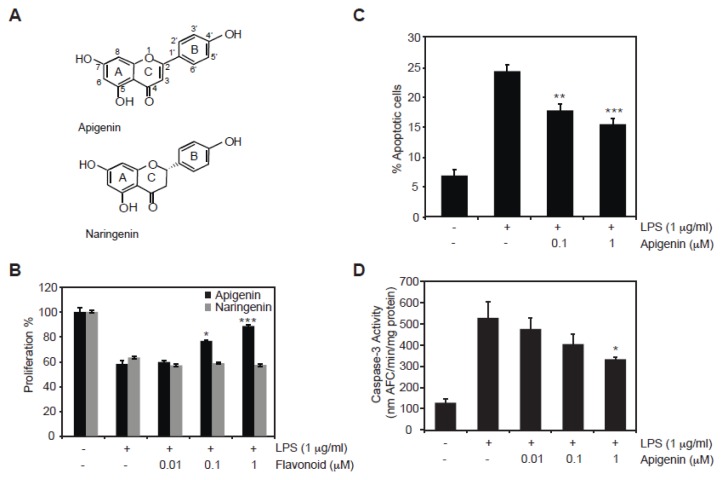
Apigenin inhibits lipopolysaccharide (LPS)-induced cell death in endothelial cells. Bovine aortic endothelial cells (BAECs) were treated with different concentrations of apigenin or naringenin ranging from 0.01, 0.1 or 1μM, or diluent dimethyl sulfoxide (DMSO) control [referred as (−)] for 30 min prior to the addition of 1 μg/mL LPS (+) for 24 h. (**A**) Chemical structures of the flavonoids apigenin and naringenin; (**B**) Percentage of cell proliferation was determined by the MTS assay; (**C**) The percentage of apoptotic cells was determined by staining cells with annexin V/7-AAD and analyzed by flow cytometry; (**D**) Caspase-3 activity was determined by the DEVD-AFC assay. Data represents mean ± SEM, *n* = 3. *p < 0.05, **p < 0.01, ***p < 0.001 determined by one-way ANOVA.

To investigate the role of apigenin on LPS-induced endothelial cell damage, the effect of apigenin in proliferation of bovine aortic endothelial cells (BAECs) was examined. BAECs were treated for 30 min with different doses of apigenin or naringenin, a related flavonoid differing only by a single double bond in ring C (Figure 1A), prior to the addition of 1 μg/mL LPS for an additional 24 h. The addition of LPS resulted in a 40% decrease of cell proliferation, as previously reported [27], compared with cells treated with the diluent DMSO (control, Figure 1B). BAECs treated for 30 min with 0.01, 0.1 and 1 μM of apigenin prior to the addition of LPS, resulted in a significant dose-dependent increase in cell proliferation compared to cells treated with LPS in the presence of diluent (Figure 1B, black bars). This effect is specific to apigenin as naringenin failed to overcome the reduction in cell proliferation induced by LPS (Figure 1B, gray bars).

Next, we investigated the effect of apigenin on LPS-induced apoptosis. LPS induced a 5-fold increase in apoptotic BAECs compared to cells treated with diluent DMSO (Figure 1C). Pre-treatment of BAECs for 30 min with 0.1 μM or 1 μM apigenin prior to the addition of LPS for 24 h resulted in a dose dependent decrease in apoptosis as demonstrated by the decrease of cells stained with annexin V/7-AAD (Figure 1C). Apoptosis induced by LPS is mediated by the activation of caspase-3, a key apoptotic effector [28]. Caspase-3 activity was barely detectable in BAECs treated with DMSO. In contrast, cells treated with 1 μg/mL LPS for 24 h showed a significant 5-fold increase in caspase-3 activity (Figure 1D). Pre-treatment with 0.01 and 0.1 μM apigenin reduced, although not significantly, LPS-mediated induction of caspase-3 activity, whereas 1 μM apigenin showed a significant decrease in LPS-induced caspase-3 activity compared to cells treated with LPS alone (Figure 1D). Collectively, these results indicate that apigenin inhibits LPS-induced apoptosis in endothelial cells.

### 2.2. Apigenin Protects from LPS-Induced Mitochondrial Dysfunction

**Figure 2:**
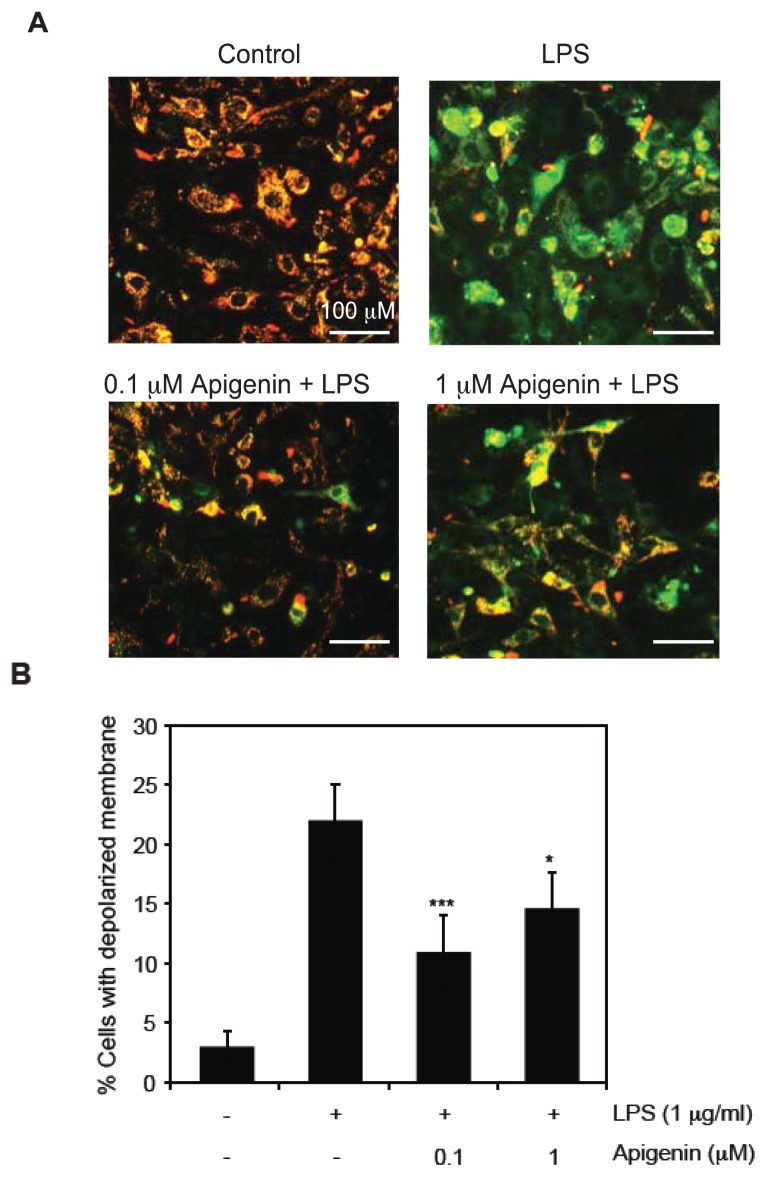
Apigenin protects LPS-induced mitochondrial depolarization in endothelial cells. BAECs were treated with 0.1 or 1 μM apigenin or diluent DMSO (−) 30 min prior to the addition of 1 μg/mL LPS (+) for 6 h. Cells treated with DMSO alone were referred as control. (**A**) Mitochondrial membrane potential was determined by fluorescence microscopy in cells stained with JC-1, representative of four independent experiments; (**B**) Flow cytometry analyses of the percentage of cells showing mitochondrial depolarization. Data represents mean ± SEM, *n* = 5. *p < 0.05, ***p < 0.001 determined by one-way ANOVA.

To understand the mechanisms involved in the ability of apigenin to reduce apoptosis during LPS-induced inflammation, we analyzed mitochondrial membrane potential (MMP). BAECs were treated with 0.1 or 1 μM apigenin or diluent DMSO 30 min prior to the addition of 1 μg/mL LPS for 6 h and MMP was determined by flow cytometry and visualized by fluorescence microscopy in cells stained with JC-1. LPS increased by 6-fold the percentage of cells with depolarized membrane, indicative of MMP loss, compared with DMSO-treated cells (Figure 2A,B). Pre-treatment of BAECs with 0.1 and 1 μM apigenin resulted in a significant decrease in the percentage of cells with depolarized membrane potential compared to cells treated with LPS alone (Figure 2A,B), indicating that apigenin prevents LPS-induced membrane depolarization and mitochondrial dysfunction.

### 2.3. Apigenin Decreases LPS-Induced ROS Production

**Figure 3:**
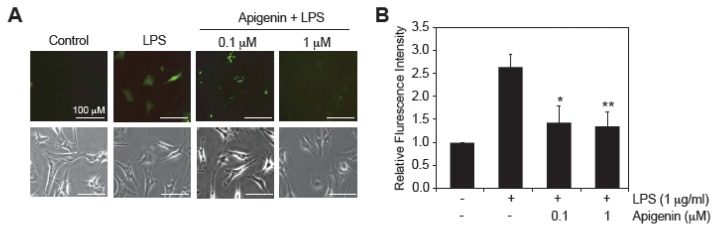
Apigenin decreases LPS-induced ROS production in endothelial cells. BAECs were treated with 0.1 or 1 μM apigenin or diluent DMSO used as control (−) 30 min prior to the addition of 1 μg/mL LPS (+) for 3 h. (**A**) Cells were stained with DCFDA and visualized under fluorescence (top) and light microscopy (bottom). Pictures are representative of four independent experiments; (**B**) Fluorescence intensity was quantified using the ImageJ software. Data represents mean ± SEM, *n* = 4. *p < 0.05, **p < 0.05 determined by one-way ANOVA.

LPS is known to exert cytotoxicity through increased ROS production in endothelial cells [7,8]. To evaluate the effect of apigenin in LPS-induced ROS production, BAECs were pre-treated with 0.1 and 1 μM apigenin for 30 min prior to the addition of 1 μg/mL LPS for an additional 3 h and the levels of ROS were measured in 2′,7′-dichlorofluorescein diacetate (DCFDA) stained cells using fluorescence microscopy (Figure 3A). LPS treatment increased 2.6-fold the levels of ROS compared to BAECs treated with diluent DMSO (Figure 3A,B). Apigenin at concentrations of 0.1 and 1 μM significantly reduced LPS-induced ROS production by 1.3 and 1.4 fold, respectively (Figure 3A,B). Taken together, these results imply that apigenin decreases LPS-induced ROS production and suggest its protective effect by preventing LPS-induced mitochondrial dysfunction in endothelial cells.

### 2.4. Apigenin Restores LPS-Induced Mitochondrial Complex I Activity

**Figure 4:**
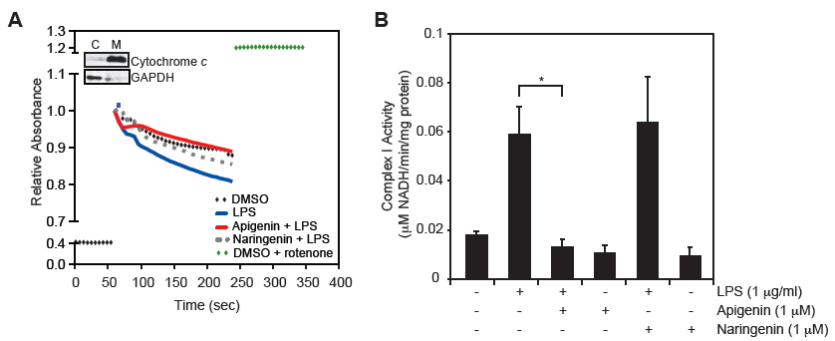
Apigenin restores LPS-induced mitochondrial Complex I activity in endothelial cells. BAECs were treated with 1 μM apigenin, naringenin or diluent DMSO 30 min prior to the addition of 1 μg/mL LPS for an additional 6 h or diluent phosphate buffered saline (PBS) (−) used as control. Complex I activity was assessed in mitochondria enriched lysates as described in Materials and Methods. (**A**) Changes in NADH absorbance at 340 nm were determined every 10 s for 6 min. Data represent average of three independent experiments. Rotenone (5 μg/mL, green diamonds), a specific inhibitor of Complex I, was added directly to the reaction to prove specificity of the assay. Inset corresponds to Western blots of cytoplasmic and mitochondria fractions probed with anti-cytochrome *c* antibodies, a mitochondrial marker and GAPDH, a cytoplasmic marker. C: cytoplasmic fraction, M: mitochondrial fraction; (**B**) Complex I activity was calculated using the enzymatic activity formula (see Materials and Methods) and expressed per minute per mg of protein. Data represent mean ± SEM, *n* = 3. *p < 0.05 determined by one-way ANOVA.

Deregulation of mitochondrial respiratory chain Complex I activity is a common cause of metabolic disorders [8,29,30]. In an attempt to understand the mechanisms underlying the protective effects of apigenin on BAECs, we examined whether apigenin could modulate Complex I activity following LPS stimulation. In order to measure Complex I activity, enriched mitochondrial fractions were obtained, as shown by immunoblotting using anti-cytochrome *c* antibody, a well-known mitochondrial marker (Figure 4A, Inset) [31]. Mitochondrial lysates from BAECs treated with 1 μM apigenin, or diluent DMSO 30 min prior to the addition of 1 μg/mL LPS for 6 h were obtained and used to determine Complex I activity by following changes in NADH absorbance over time. Rotenone (5 μg/mL), a specific Complex I inhibitor, when added directly to the mitochondrial-enriched lysates resulted in the complete inhibition of NADH oxidation (Figure 4, green diamonds), supporting the specific evaluation of Complex I using this approach. BAECs treated with LPS showed a decrease in NADH absorbance (Figure 4A, blue line), corresponding to a 3-fold increase in complex I activity (Figure 4B), compared to cells treated with diluent DMSO (Figure 4A, black diamonds; Figure 4B, marked [−/−/−]). Pre-treatment with 1 μM apigenin restored LPS-induced Complex I activity to levels found in cells treated with diluent DMSO (Figure 4A, red line; Figure 4B). In contrast, naringenin had no effect on the complex I activity of LPS-treated cells (Figure 4A, gray dotted line; Figure 4B). Complex I activity in BAECs treated with 1 μM apigenin or naringenin alone for 6 h was similar to control (Figure 4B). Taken together, these results indicate that LPS impacts the mitochondrial respiratory chain in endothelial cells, hence increasing the production of ROS and leading to membrane depolarization and apoptosis. In contrast, apigenin restores Complex I activity to normal levels, thus balancing the deleterious effect of LPS on mitochondrial function.

### 2.5. Discussion

Flavonoids are emerging as potent alternative anti-inflammatory agents. However, their mechanisms of action remain poorly defined. Previously, we reported that apigenin completely halts LPS-induced mortality by decreasing inflammatory cytokine production through modulation of NF-κB activity [19]. Despite these findings, the underlying mechanisms of apigenin-induced survival during septic shock remain elusive. The present study demonstrates that apigenin exerts anti-inflammatory activity by reducing LPS-induced ROS production and apoptosis, and balancing mitochondrial function in LPS-treated endothelial cells.

The pathogenesis of sepsis is yet viewed as a complex network of events, where the endothelium plays a central role in microvascular dysfunction [9]. LPS is the predominant exogenous mediator of sepsis and is known to induce significant damage to vascular endothelium through generation of ROS and mitochondria dysfunction leading to metabolic disorder and eventually organ failure [8,9]. Thus, reestablishment of metabolic balance during sepsis by apigenin constitutes an attractive strategy for preserving organ function during inflammatory diseases.

Apoptosis of endothelial cells is a characteristic of sepsis [32,33]. Our results show that apigenin can considerably improve endothelial cell viability when administered prior to LPS, by reducing caspase-3 activity and apoptosis in LPS-treated BAECs (Figure 1), indicating a cytoprotective role of apigenin towards LPS. To evaluate specificity, we used naringenin, a flavonoid with similar chemical structure that lacks anti-inflammatory activity. Naringenin had no effect, suggesting that the protective role of apigenin observed here is a unique characteristic of this particular flavonoid (Figure 1).

LPS is known to induce copious generation of ROS, primarily superoxide anion (O_2_^−^), hydrogen peroxide (H_2_O_2_) and peroxynitrite [14], although the mechanisms are not fully understood. Elevated ROS production leads to loss in MMP, causing endothelial cell death [14]. Maintenance of the MMP is crucial to metabolic homeostasis [34,35]. Studies in sepsis suggest that changes in mitochondrial membrane integrity mediate multiple organ failure and correlates with the severity of the disease [36,37]. Congruent with the current literature, our findings confirmed that LPS induces membrane depolarization and increases ROS production in endothelial cells. Moreover, our results indicate that apigenin at concentrations of 0.1 or 1 μM was able to restore LPS-induced mitochondrial membrane depolarization (Figure 2) and attenuated LPS-induced ROS production (Figure 3). Our results support a model in which apigenin, exerts its anti-apoptotic function by normalizing mitochondria function in LPS-treated endothelial cells.

**Figure 5:**
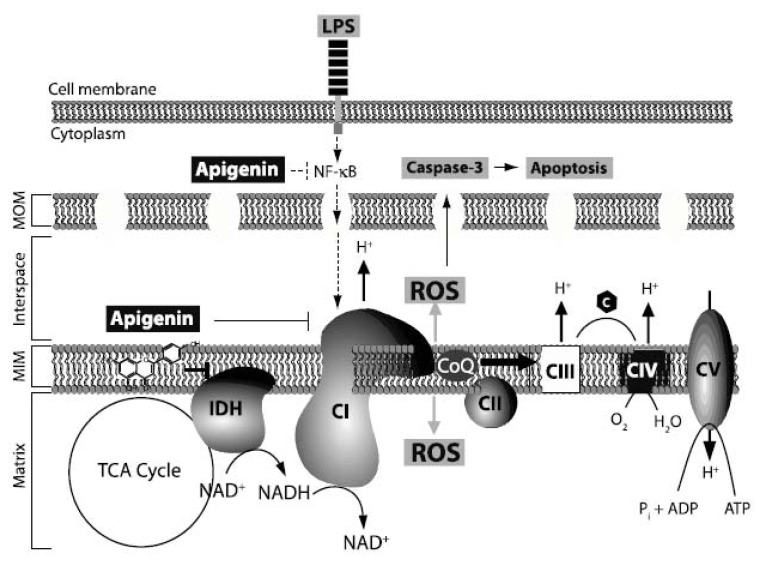
Schematic representation of the mechanisms by which apigenin may modulate LPS-induced mitochondrial dysfunction. LPS increases the NADH dehydrogenase activity of Complex I (CI), ROS production, mitochondrial membrane depolarization and apoptosis. Apigenin decreases the LPS-induced activity of Complex I thus balancing the deleterious effect of LPS on mitochondrial function and rescuing cells from LPS-induced apoptosis. MOM: mitochondria outer membrane; MIM: mitochondria inner membrane; c: cytochrome *c*; CoQ: Ubiquinone. Complexes II, III, IV and V (ATP synthase) and IDH (isocitrate dehydrogenase) are shown. CI, CIII and CIV are proton-pumping enzymes.

The mitochondrial respiratory chain (electron transport chain), which includes four individual enzyme complexes (Figure 5), *i.e.*, NADH-ubiquinone oxidoreductase (Complex I), succinate dehydrogenase (Complex II), Ubiquinone-cytochrome *c* reductase (Complex III) and cytochrome *c* oxidase (Complex IV), is considered the powerhouse of cells, generating more than 90% of energy in the form of ATP [38]. Its malfunction leads to increased ROS production and metabolic disorder. Mitochondrial function during sepsis and endotoxin shock has been extensively investigated. Current literature presents conflicting findings regarding effects of LPS on mitochondrial respiration. While some studies have reported that LPS decreases the transfer of electron from Complex I to Complex III [39–41], others have shown that LPS increases mitochondria respiratory chain [42–44]. For example, Vanasco and co-workers [41] found decreased cardiac mitochondrial function, while others [42], reported increased hepatic mitochondrial function in animal models of sepsis. These disagreements seem to be tissue/cell type dependent. Recent exciting findings, during the preparation of this manuscript, suggest the existence of mitochondrial respiratory super-complexes, providing a novel view of the mitochondrial respiration plasticity [45]. Thus, whether Complex I itself or by directly associating with Complex III or IV regulate mitochondrial function in disease, will need to be re-evaluated [45]. Regardless of these discrepancies, there is consensus that severe sepsis impacts the mitochondrial respiratory chain leading to ROS production and membrane depolarization. However, while the recognition of the central role of metabolism in the pathophysiology of inflammatory diseases is gaining momentum, the mechanisms underlying inflammation-mediated mitochondrial dysfunction are yet to be elucidated. Our results showed that LPS significantly augmented the NADH dehydrogenase activity of Complex I in endothelial cells, whereas apigenin reduced this activity to levels found in control cells (Figure 4). The basic mechanisms associated with these observations are yet to be determined and whether apigenin inhibits LPS-induced Complex I activity or super-complexes formation will need further evaluations. Recent studies showed that NF-κB activity promotes mitochondrial respiration [46]. Our previous studies showed that apigenin inhibited LPS-induced NF-κB activity [19,20]. Therefore, it is conceivable that apigenin by inhibiting NF-κB activity counteracts the effect of LPS on mitochondrial function (Figure 5).

In addition, we previously reported that apigenin localizes to the mitochondria [25] and that apigenin inhibits the NAD^+^ dependent isocitrate dehydrogenase 3 (IDH3) activity [26], a major regulatory point in the tricarboxylic acid (TCA) cycle [47]. Thus, an additional possibility is that apigenin, by halting IDH activity, interferes with mitochondrial respiration (Figure 5). Interestingly, it was previously shown that apigenin, and other flavonoids, including quercetin and kaempferol, decreased Complex I activity in isolated mitochondria from rat liver [48,49]. Hence, it is also plausible that apigenin exerts its effects by directly affecting a component of Complex I and thus decreasing its activity on LPS-treated cells (Figure 5). It remains to be established if the properties of apigenin in balancing mitochondrial function can be extended to other tissue/cell type or pathologies with high mitochondrial activity.

Collectively, the present study provides evidence pertaining to the protective role of apigenin in LPS-induced cytotoxicity and mitochondrial dysfunction in endothelial cells and showcases the promising role of apigenin in improving metabolic parameters in inflammatory conditions such as sepsis. Our results contribute to a better understanding of the anti-inflammatory mechanisms of the dietary flavonoid apigenin and suggest the possibility that apigenin prevents sepsis-induced mortality by decreasing endothelium cell death.

## 3. Experimental Section

### 3.1. Chemicals, Cell Lines and Cultures

Bovine aortic endothelial cells (BAECs) were purchased from Cell Systems (Kirkland, WA, USA) and cultured as previously described [50]. DEVD-AFC was purchased from Enzyme System Product (Livermore, CA, USA). Dulbecco’s Modified Eagle’s Medium (DMEM), Fetal Bovine Serum (FBS), penicillin-streptomycin, trypsin, 2′,7′-dichlorofluorescein diacetate (DCFDA) and 5,5,6,6-tetrachloro-1,1,3,3-tetraethylbenzimidazol-carbo-cyanine iodide (JC-1) were purchased from Life Technologies (Carlsbad, CA, USA). PE-Annexin V Apoptosis Detection Kit was from BD Biosciences (Franklin Lakes, NJ, USA). Apigenin, naringenin, diluent dimethyl sulfoxide (DMSO), lipopolysaccharide (LPS, *Escherichia coli* serotype 0127:B8), NaN_3_, sodium cholate, β-nicotinamide adenine dinucleotide, reduced (NADH), rotenone and ubiquinone-1 (CoQ_1_) were purchased from Sigma (St. Louis, MO, USA).

### 3.2. Cell Viability Assay (MTS Assay)

BAECs were plated at a density of 8000/100 μL in 96-well plates. After 24 h, BAECs were treated with 0.01, 0.1 or 1 μM apigenin, naringenin or DMSO, 30 min prior to treatment with 1 μg/mL LPS for 24 h and cell viability was determined by MTS assay using the CellTiter 96 Aqueous One Solution according to manufacturer’s protocol (Promega, Madison, WI, USA). Absorbance at 490 nm was measured using the EnSpire multimode plate reader (PerkinElmer, Waltham, MA, USA).

### 3.3. Determination of Caspase-3 Activity

BAECs were treated with 0.01, 0.1 or 1 μM apigenin or DMSO (control) for 30 min prior to incubation with LPS (1 μg/mL) for 24 h. Caspase-3 activity was determined in BAEC lysates using the DEVD-AFC assay, as previously described [51,52]. Released AFC was measured using a Cytofluor 4000 fluorometer (filters: excitation, 400 nm; emission, 505 nm; Perceptive Co., Framingham, MA, USA).

### 3.4. Detection of Apoptosis

BAECs were treated with 0.1 or 1 μM apigenin or DMSO (control) for 30 min prior to incubation with LPS (1 μg/mL) for 24 h and subsequently stained with PE-conjugated Annexin V (1 μg/mL), 7-AAD (0.5 μg/mL) according to manufacturer’s suggestions (BD Biosciences, San Jose, CA, USA). Percentage of apoptosis was determined using a BD FACSCalibur (BD Biosciences, San Jose, CA, USA) and data were analyzed using FlowJo research software (BD Biosciences, San Jose, CA, USA).

### 3.5. Determination of Mitochondrial Membrane Potential (MMP)

BAECs were treated with 0.1 or 1 μM apigenin or DMSO (control) for 30 min prior to incubation with LPS (1 μg/mL) for 6 h. BAECs were rinsed twice with phosphate buffered saline (PBS), followed by incubation with 1 μg/mL JC-1 for 30 min at 37 °C in the dark and subsequently washed twice with PBS. Changes in MMP were determined using BD LSR II flow cytometer (BD Biosciences, San Jose, CA, USA) by measuring the fluorescence intensity in both the FL-2 transmits 585 nm and FL-1 transmits 530 nm channels. The percentage of mean fluorescence intensity (% MFI) was determined and the percentage of cells with depolarized membrane calculated using FlowJo research software (BD Biosciences, San Jose, CA, USA).

### 3.6. Detection of Reactive Oxygen Species (ROS)

BAECs were treated with 0.1 or 1 μM apigenin or DMSO (control) for 30 min prior to incubation with LPS (1 μg/mL) for 3 h. Next, BAECs were collected, washed with PBS three times, followed by incubation with 25 μM DCFDA for 30 min at 37 °C in the dark. ROS generation was assessed using the fluorescence microscope (Olympus BX40 equipped with the Optronics DEI 750E CE Digital Camera, Olympus, Center Valley, PA), as previously described [53]. Fluorescence intensities of cells from different fields were measured using the ImageJ software (NIH, Bethesda, MD, USA).

### 3.7. Complex I Activity Assay

Complex I activity was determined in mitochondrial fractions from BAECs treated with 1 μM apigenin, 1 μM naringenin or diluent DMSO for 30 min prior to treatment with 1 μg/mL LPS for 6 h. Mitochondria were isolated from 30 × 10^6^ BAECs by dounce homogenization (100 strokes) in 400 μL of mitochondria isolation (MI) buffer containing 0.25 M sucrose, 2 mM EGTA, 20 mM Tris-HCL pH 7.6, 20 mM HEPES, 1 mg/mL BSA, 0.1 mM PMSF, 2 μg/mL each of chymostatin, pepstatin, antipain, and leupeptin, and centrifuged at 1500 *g* for 10 min at 4 °C. Pellets were resuspended in 400 μL MI buffer and centrifuged at 17,000 *g* for 30 min at 4 °C. Next, pellets containing mitochondrial fraction were resuspended in 200 μL MI buffer and lysed by three rounds of freeze and thaw cycles. Purity of the isolated mitochondrial fraction was verified by Western blot using antibodies against cytochrome *c*, a specific mitochondrial marker, and GAPDH, a cytoplasmic marker. Complex I activity was evaluated by incubating 50 μg of mitochondrial protein in 200 μL buffer containing 20 mM potassium phosphate buffer pH 7.6, 2 mM NaN_3_, 0.8% sodium cholate, and 16 mM ubiquitin. The reaction was initiated by the addition of 1.5 mM NADH. Rotenone (5 μg/mL), a specific inhibitor of Complex I, was added directly to the reaction to prove specificity of the assay. Activity was determined by assessing the change in NADH absorbance at 340 nm every 10 s for 6 min using the EnSpire multimode plate reader (PerkinElmer, Waltham, MA, USA). Enzymatic units were calculated using the following formula [54]:

(1) $$\text{Enzymatic units}=(\Delta {\text{A}}_{340}V_{\text{f}}d.f.)/ɛmgl)$$

where ΔA_340_ is the change in absorbance at 340 nm over time; *V*_f_ is the final reaction volume; *d.f.*, dilution factor; ɛ, extinction coefficient of NADH determined to be 6.22; mg, amount of protein; and *l*, path length estimated to be 0.68 cm.

### 3.8. Statistical Analysis

All results are represented as mean ± standard error of mean (SEM) from three, four or five independent experiments. Statistical analyses of data were done by one-way analysis of variance (ANOVA) followed by Bonferroni’s post hoc comparisons using GraphPad Prism (GraphPad Software, San Diego, CA, USA). A *p-*value < 0.05 was considered statistically significant.

## 4. Conclusions

In summary, our findings suggest mitochondria as a plausible target where the flavonoid apigenin imparts its protective effects against LPS-induced mitochondrial damage. The findings are of clinical relevance, as it opens up avenues for the formulation of novel nutraceutical therapy in treating inflammatory diseases induced by deregulation of the mitochondria.
